# Meningeal retinoic acid contributes to neocortical lamination and radial migration during mouse brain development

**DOI:** 10.1242/bio.021063

**Published:** 2016-12-23

**Authors:** Carole Haushalter, Brigitte Schuhbaur, Pascal Dollé, Muriel Rhinn

**Affiliations:** 1 Development and Stem Cells Department, Institut de Génétique et de Biologie Moléculaire et Cellulaire, Illkirch 67404, France; 2 Centre National de la Recherche Scientifique, UMR 7104, Illkirch 67404, France; 3 Institut National de la Santé et de la Recherche Médicale, U 964, Illkirch 67404, France; 4 Université de Strasbourg, Illkirch 67404, France

**Keywords:** Retinoids, Cerebral cortex, Neurons, Radial migration, Cortical layering

## Abstract

Retinoic acid (RA) is a diffusible molecule involved in early forebrain patterning. Its later production in the meninges by the retinaldehyde dehydrogenase RALDH2 coincides with the time of cortical neuron generation. A function of RA in this process has not been adressed directly as *Raldh2*^−/−^ mouse mutants are embryonic lethal. Here, we used a conditional genetic strategy to inactivate *Raldh2* just prior to onset of its expression in the developing meninges. This inactivation does not affect the formation of the cortical progenitor populations, their rate of division, or timing of differentiation. However, migration of late-born cortical neurons is delayed, with neurons stalling in the intermediate zone and exhibiting an abnormal multipolar morphology. This suggests that RA controls the multipolar-to-bipolar transition that occurs in the intermediate zone and allows neurons to start locomotion in the cortical plate. Our work also shows a role for RA in cortical lamination, as deep layers are expanded and a subset of layer IV neurons are not formed in the *Raldh2*-ablated mutants. These data demonstrate that meninges are a source of extrinsic signals important for cortical development.

## Abstract (precis)

**Summary:** Involvement of the signalling molecule retinoic acid in neurogenesis of the developing cerebral cortex is shown through targeted deletion of its synthesizing enzyme.

## INTRODUCTION

Early induction events in the anterior neural plate define the embryonic forebrain. One of its derivatives is the cerebral cortex, a brain center responsible for the control of higher cognitive functions, perceptions and emotions. At the beginning of corticogenesis, neuroepithelial (NE) cells give rise to radial glial (RG) cells (also called apical progenitors) located in the ventricular zone (VZ), which undergo symmetrical and proliferative divisions to self-renew. As development proceeds, RG cells divide asymmetrically to produce post-mitotic neurons or intermediate neuronal progenitor (INP) cells. INP cells localise in the subventricular zone (SVZ), divide a few times (1-2), and differentiate into neurons (Götz and Huttner, 2005; Miyata et al., 2010; Noctor et al., 2004). Newborn neurons migrate through the intermediate zone (IZ) and eventually give rise, in an inside-out manner, to five cortical layers known as layers II to VI (with layer I being essentially composed of axons and dendritic tufts) (Rakic, 1974; Sidman and Rakic, 1973). Neurons produced during early neurogenesis [embryonic day (E)12.5-13.5 in mouse] generate deeper layers of the cortex (layers VI and V), whereas those born later generate the more superficial (upper) layers II/III and IV (Polleux et al., 1997). To reach their destination in the cortical plate, early-born neurons move by somal translocation (Nadarajah et al., 2001), whereas neurons born at E14.5 and later use glia-guided locomotion to migrate (LoTurco and Bai, 2006). While traveling through the SVZ and IZ, the late-born neurons acquire a transient multipolar morphology by sprouting out multiple neurites (LoTurco and Bai, 2006; Noctor et al., 2004; Tabata and Nakajima, 2003). In the upper IZ, they acquire a bipolar morphology allowing a proper glia-guided locomotion. Eventually, to reach their final position, they extend their leading processes to the pial surface and switch to a glia-independant somal translocation (Nadarajah et al., 2001). Migration of late-born neurons is also regulated by the secretion of the extracellular protein Reelin from Cajal–Retzius cells (Inoue et al., 2008), which are the first neurons to be born. They originate at three focal points in the embryonic telencephalon (the cortical hem, the septum and the ventral pallium) and spread tangentially to cover the cortical plate, thus forming layer I (Borrell and Marín, 2006).

A tight control of all these coordinated steps is required during early corticogenesis and involves specific transcription factors, as well as signalling molecules (for review, see Miyata et al., 2010). One candidate signal is retinoic acid (RA), a key regulator of several processes during development and organogenesis (Rhinn and Dollé, 2012 for a review). RA is a lipophilic molecule that acts a diffusible ligand for nuclear receptors, RARs (retinoic acid receptors) and RXRs (retinoid X receptors), thereby controlling the transcription of many genes. The presence of RA in specific embryonic tissues depends on its regulated synthesis. In the embryo, vitamin A or retinol is uptaken transplacentally and is transformed into RA in a two step oxydation involving retinol dehydrogenases (mainly RDH10) and then retinaldehyde dehydrogenases (RALDH1/2/3). During head development, *Raldh2* is the first enzyme­-encoding gene to be expressed at E8 within the anterior forebrain neuroepithelium and the overlying surface ectoderm. Progressively, *Raldh2* expression recedes from the forebrain neuroepithelium, while surface ectoderm expression persists until E9.5 (Niederreither et al., 1999; Ribes et al., 2006). Thus, until E9.5, RALDH2 is responsible for RA signalling in the embryonic head and our analysis of *Raldh2^−/−^* mutant mice suggests that this peak of RA occuring at E8.5 acts on the growth and organisation of anterior neural tissue (Niederreither et al., 1999; Ribes et al., 2006). Interestingly, *Raldh2* shows a second peak of expression in the meninges surrounding the cerebral cortex, starting at E12.5 ventrally and encompassing the whole meninges by E14.5 (Siegenthaler et al., 2009; Smith et al., 2001). Thus, RALDH2 activity may define a source of RA in the meningeal space, which could act on the underlying cortex where it may influence neurogenesis. Indeed, a number of studies have shown that the retinoid pathway acts on neuronal differentiation, proliferation, neurite outgrowth, and synaptogenesis (Corcoran et al., 2000; Maden, 2007, 2015; McCaffery et al., 2003, and references therein). As *Raldh2^−/−^* mutants are early embryonic lethal at E9.5 (Niederreither et al., 1999), a possible function of RA at later stages of cortical neurogenesis has not been investigated directly. In this study, we have analyzed the development of the cerebral cortex in embryos lacking RA produced by RALDH2 in the developing meninges. Using a *Raldh2* conditional knockout (Raldh2cKO), we showed that loss of function of *Raldh2* from the beginning of its expression in the meninges did not affect the formation of progenitor cells, including RG and INP cells, nor the birth of newborn neurons. However, we observed an abnormal layering of cortical neurons, mainly affecting layer IV. Also, by tracing the newborn neurons using *in utero* electroporation of a GFP marker, we showed that loss of RA in the developing cerebral cortex transiently affected the migration of newborn neurons.

## RESULTS

### Conditional deletion of *Raldh2*

**Fig. 1.**
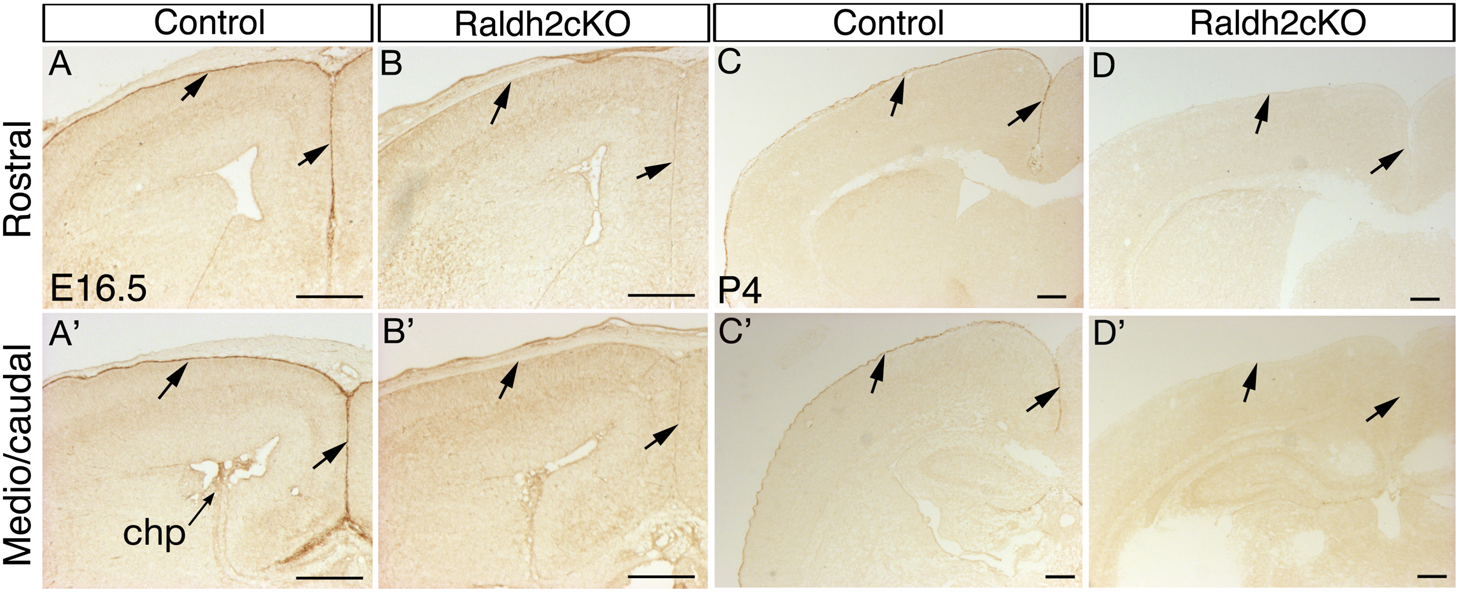
**Tamoxifen-induced ablation of *Raldh2* in the developing meninges.** (A,A′,C,C′) Immunodetection of RALDH2 on coronal sections of the brain of a control (*Raldh2^flox/flox^;CMV-βactin-Cre-ERT2*^0^) mouse at E16.5 (A,A′) and at P4 (C,C′). Sections are shown at a rostral (A-D) and more caudal (A′-D′) level of the brain, showing RALDH2 expression in the meningeal layer overlying the cerebral cortex (arrows), and in choroid plexus (chp). (B,B′,D,D′) Comparative views of the brain of a Raldh2cKO (*Raldh2^flox/flox^; CMV-βactin-Cre-ERT2*^+^) mutant, showing absence of RALDH2 signal. Tamoxifen was administered at E10.5. Scale bars: 250 μm.

After its transient early expression in forebrain neuroepithelial cells and surface ectoderm (Ribes et al., 2006), *Raldh2* is expressed starting at E12.5 in the lateral and medial meninges and is present in the entire meninges by E14.5-16.5 (Siegenthaler et al., 2009) (Fig. 1A,A′). Until birth, the meninges are the only brain structure where RALDH2 can be detected (Smith et al., 2001) (Fig. 1C,C′ and data not shown).

To address the function of RA produced by the meninges, it was necessary to overcome the early embryonic letality of germline *Raldh2^−/−^* mutants. We used the *Cre-ERT2* recombinase system to induce a temporally controlled deletion of *Raldh2* by tamoxifen induction. A *Raldh2* conditional knockout mouse line that was previously described (*Raldh2^flox/flox^*; Vermot et al., 2006) was crossed with the *CMV-βactin-Cre-ERT2* transgenic line (Santagati et al., 2005). For tamoxifen induction, 10 mg of tamoxifen was given to pregnant females at E10.5 by oral gavage. The loss of RALDH2 in tamoxifen-induced (Raldh2cKO) mutants was validated by immunolabellings on E16.5 brains (Fig. 1A-B′) and postnatal day (P)4 brains (Fig. 1C-D′). When compared to control littermates (devoid of the *Cre-ERT2* transgene), Raldh2cKO animals did not display any detectable morphological abnormality at prenatal stages, and upon dissection their brains were comparable to those of control mice (data not shown).

### Integrity of the meninges and cortical marginal zone upon deletion of *Raldh2*

**Fig. 2.**
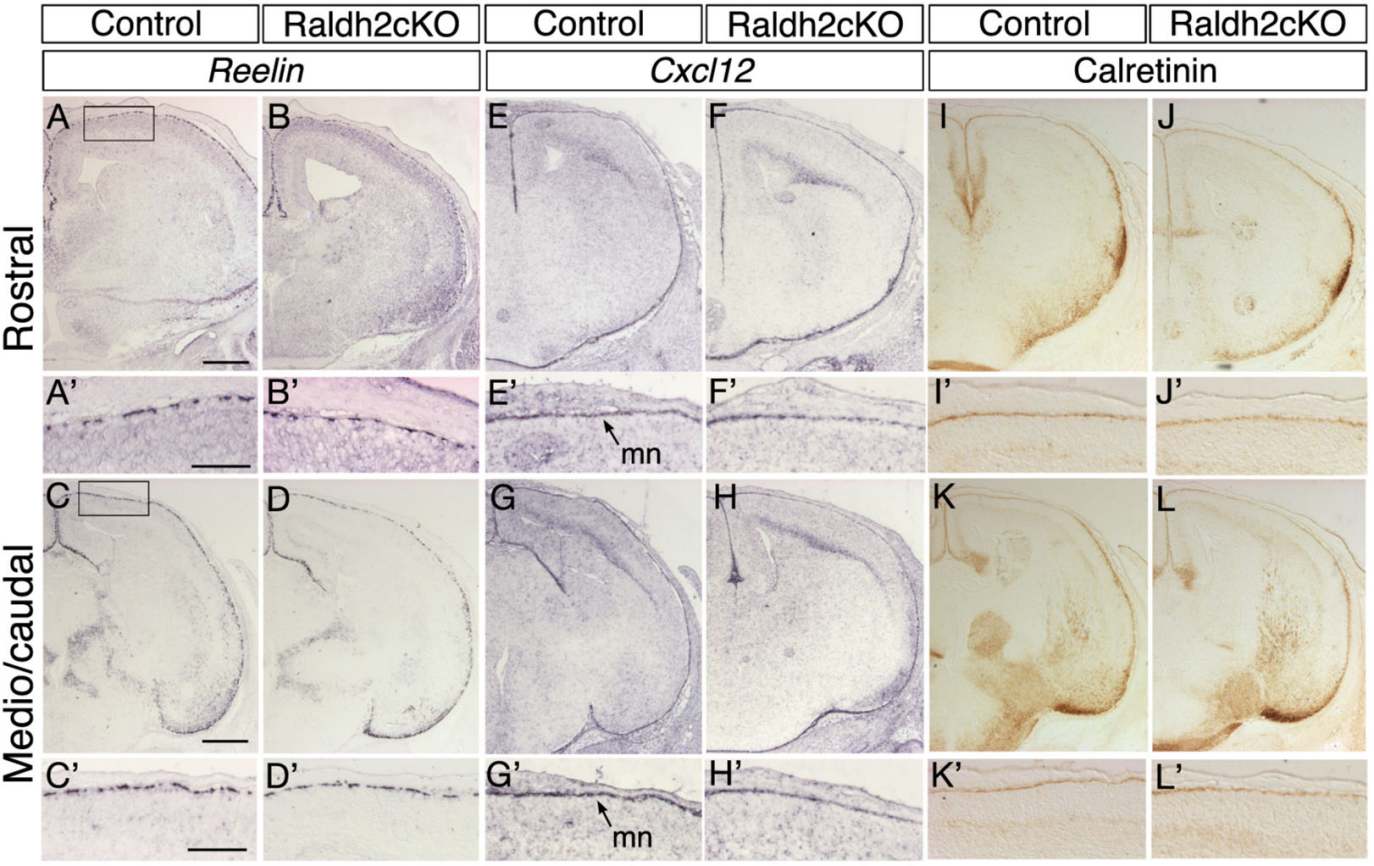
**Analysis of Cajal–Retzius cells and developing meninges in Raldh2cKO mice.** Comparative, coronal E16.5 brain sections are shown at two levels, rostral (upper panels) and more caudal (lower panels). *In situ* hybridisation for *Reelin* (A-D′) and immunolabellings for Calretinin (I-L′) show that their distribution in Cajal–Retzius cells of the cortical marginal zone of control embryos is unchanged in Raldh2cKO embryos. *In situ* hybridisation for *Cxcl12* (E-H′), a marker of the developing meninges, also shows a normal distribution in Raldh2cKO embryos. mn, meninges. Scale bars: 500 μm (A-L), 250 μm (A′-L′).

Cajal–Retzius (CR) cells are a transient population of neurons located in the marginal zone (MZ) of the developing cerebral cortex, beneath the meninges (del Río et al., 1995; Derer, 1985). They play a crucial role for cortical lamination as they regulate migration and final positioning of neurons in the cortical plate via the secretion of an extracellular glycoprotein, Reelin, in a process called glia-independant somal translocation (Dulabon et al., 2000; Hartfuss et al., 2003). Integrity of the meninges is critical for the correct spread and final localisation of CR cells (Inoue et al., 2008). Cxcr4, a chemokine receptor, is expressed by CR cells (Stumm et al., 2003) and its ligand, Cxcl12, is expressed in the meninges. Through their interaction, both proteins play a crucial role in the positioning of CR cells in the MZ (Borrell and Marín, 2006). We analysed by *in situ* hybridisation the expression of *Reelin* (CR cells; Fig. 2A-D′) and *Cxcl12* (meninges; Fig. 2E-H′) in control and Raldh2cKO mice at E16.5, and observed comparable distributions of labelled cells in both genotypes. Furthermore, using immunohistochemistry we analysed the distribution of the calcium-binding protein Calretinin, another marker of CR cells. Again, there was no detectable difference between control and Raldh2cKO mice (Fig. 2I-L′), and examination at high magnification showed an almost continous band of *Reelin-* and Calretinin-labelled CR cells along the cortical marginal zone in both genotypes (Fig. 2A-D′,I-L′). Also, examination at higher magnification of *Cxcl12* expression did not reveal any differences between control and Raldh2cKO mice (Fig. 2E-H′), indicating the presence of a functional meningeal layer – with respect to its signalling towards CR cells – in the absence of RALDH2.

### Absence of RALDH2 leads to abnormal neuronal layers in the cerebral cortex

Laminar-specific cortical neurons can be distinguished through the expression of distinct (layer-specific) transcription factors that control cortical neuronal identities and properties (see Molyneaux et al., 2007 for a review). To investigate the requirement of RA in layer-specific cortical neuronal differentiation, we examined expression of these molecular markers in brains of early postnatal mice. The brains of the pups were dissected at P4, and the loss of RALDH2 validated by immunolabellings (Fig. 1C-D′).

**Fig. 3.**
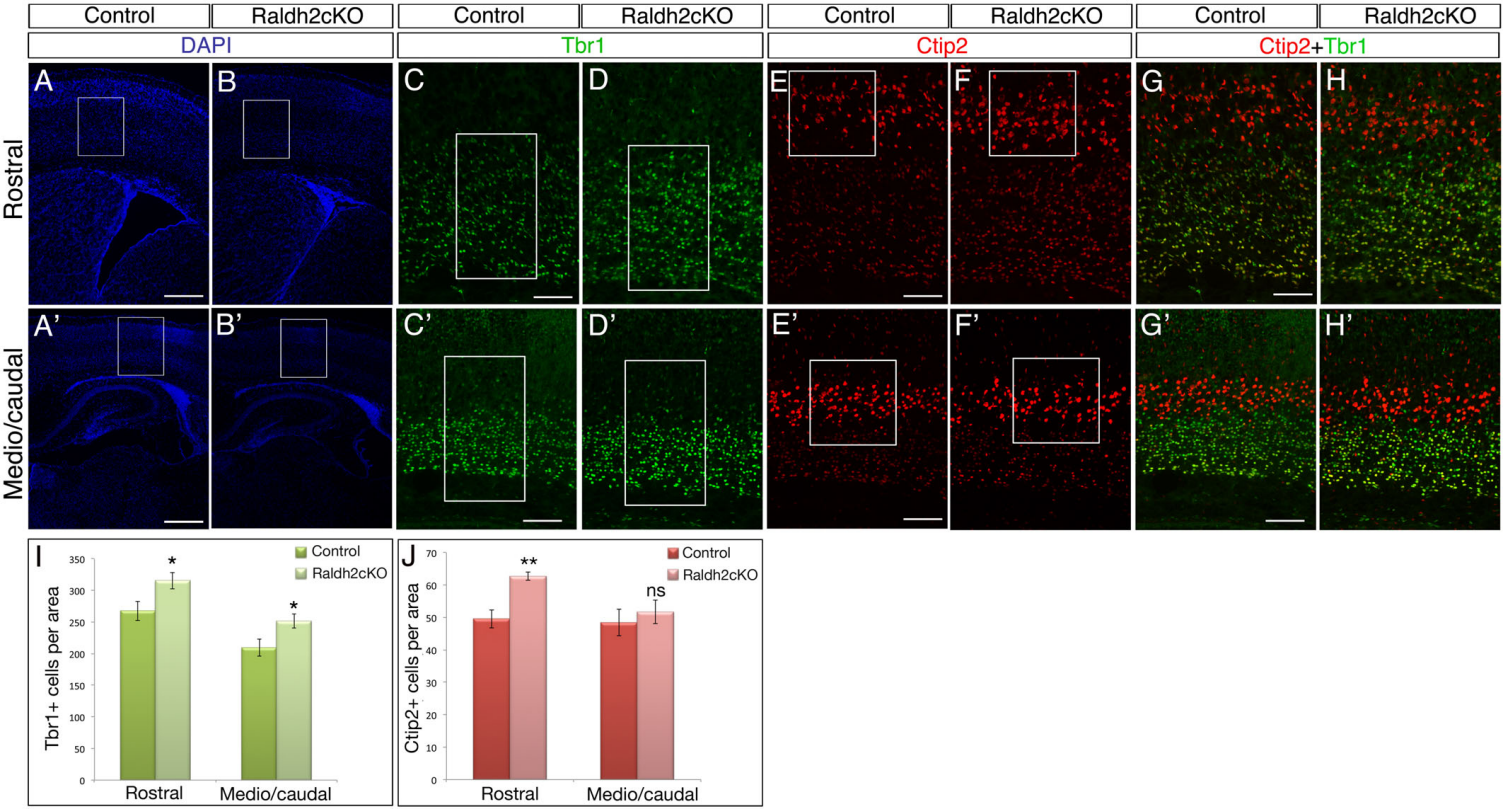
**Postnatal Raldh2cKO mice show increased numbers of neurons in deep layers of the cortex.** (A-H′) Labellings on brains sections from P4 control and Raldh2cKO animals for DAPI (A-B′), and immunolabellings for Tbr1 (C-D′) or Ctip2 (E-F′) are shown on comparative sections at rostral (A-H) and caudal (A′-H′) levels. (G-H′) Overlays of the Tbr1/Ctip2 signal. White boxes in A-B′ show the area selected for the higher magnification pictures of the Tbr1 and Ctip2 immunolabellings. White boxes in C-F′ show the areas used for cell counts. (I) Quantification of Tbr1-positive cells; rostrally: 267.6±15.00 (control) and 315.26±13.08 (Raldh2cKO); caudally: 209.66±13.88 (control) and 251.73±11.61 (Raldh2cKO). (J) Quantification of Ctip2-positive cells; rostrally: 49.6±2.79 (control) and 62.73±1.24 (Raldh2cKO); caudally: 48.46±4.1 (control) and 51.66±3.65 (Raldh2cKO). Data presented as mean±s.e.m.; *n*=5 brains; *P<0.05, **P<0.01; ns, not significant by two-tailed Student's *t*-test. Scale bars: 400 μm (A-B′), 100 μm (C-H′).

Immunolabellings of projection neurons sitting in deeper layers (DL) of the cortex were performed using Tbr1 (layer VI) (Hevner et al., 2001) and Ctip2 (layer V) (Arlotta et al., 2005). Raldh2cKO mutants showed a 18% significant increase of layer VI Tbr1-positive neurons (Fig. 3A-D′,G-H′,I; *P*<0.05), as well as a 26% significant increase of layer V Ctip2-positive neurons (Fig. 3A-B′,E-H′,J; *P*<0.01) on rostral sections of the cortex. The cell density of the layers was evaluated and was unchanged (data not shown). These data support a role for RA in establishing the DL in the cortex.

**Fig. 4.**
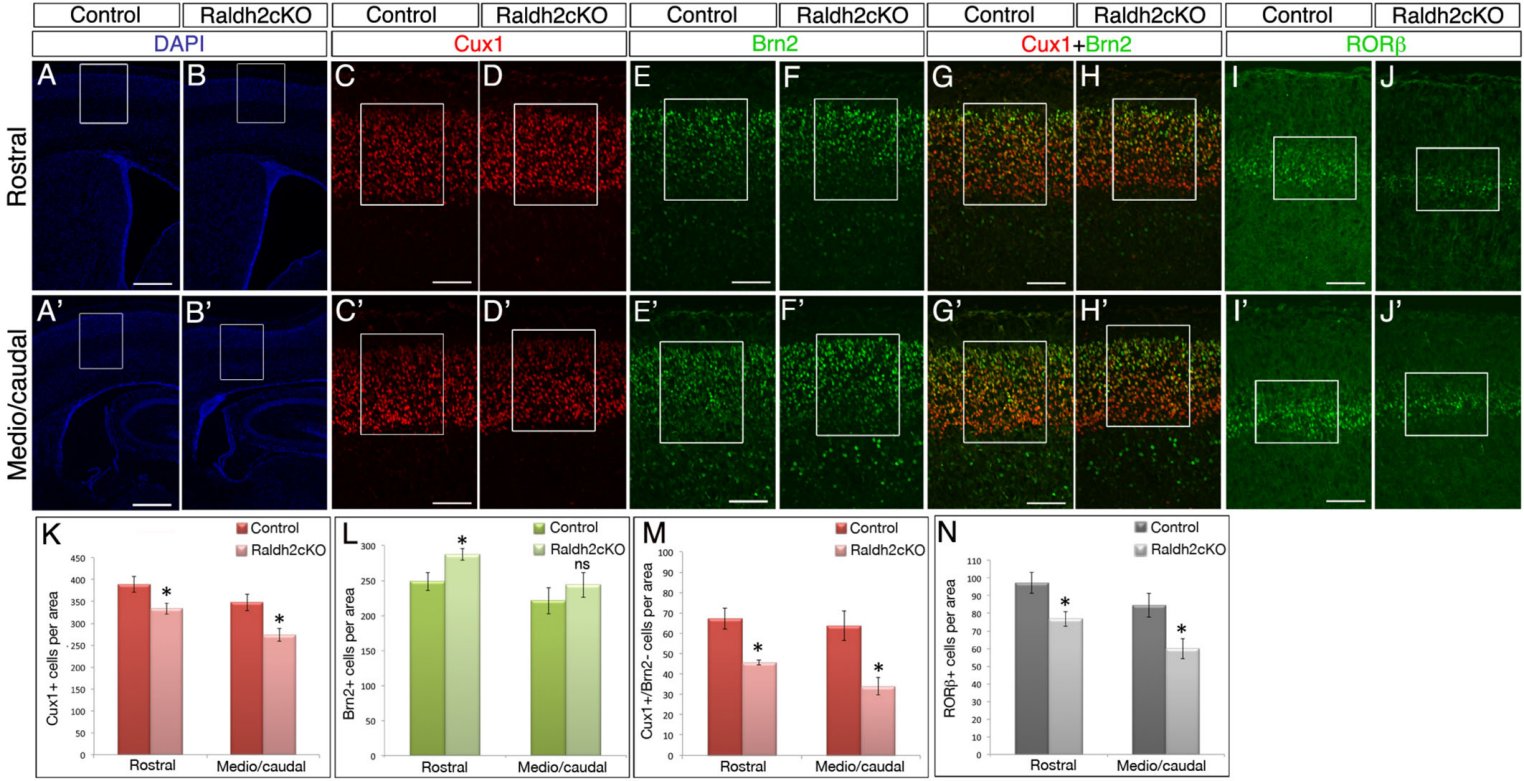
**Postnatal Raldh2cKO mice have decreased neuron numbers in upper cortical layers.** (A-J′) Labellings on brain sections from P4 control and Raldh2cKO animals for DAPI (A-B′) and immunolabellings for Cux1 (C-D′), Brn2 (E-F′) or RORβ (I-J′) at rostral (A-J) levels and caudal (A′-J′) levels. (G-H′) Overlay of Cux1 and Brn2 signals. White boxes in A-B′ show the areas selected for the higher magnification pictures of the Cux1, Brn2 and RORβ immunolabellings, and in C-J′ the areas used for cell counts. (K) Quantification of Cux1-positive cells; rostrally: 388.4±17.76 for control and 333.26±12.68 for Raldh2cKO; caudally: 347.6±18.68 for control and 273.73±15.50 for Raldh2cKO. (L) Quantification of Brn2-positive cells; rostrally: 248.26±12.60 for control and 287.2±8.22 for Raldh2cKO; caudally: 221.06±18.91 for control and 243.4±17.53 for Raldh2cKO. (M) Quantification of Cux1-positive/Brn2-negative cells; rostrally: 67.25±5.18 for control and 45.58±1.14 for Raldh2cKO; caudally: 63.66±7.25 for control and 33.83±4.27 for Raldh2cKO. (N) Quantification of RORβ-positive cells; rostrally: 97.33±5.95 for control and 76.8±4.11 for Raldh2cKO; caudally: 84.53±6.73 for control and 59.93±5.71 for Raldh2cKO. Data presented as mean±s.e.m.; *n*=5 brains; *P<0.05; ns, not significant by two-tailed Student's *t*-test. Scale bars: 400 μm (A-B′), 100 μm (C-J′).

We analysed projection neurons of upper layers (UL) by using Cux1 (layers IV-II) (Nieto et al., 2004) and Brn2 (layers II-III) (Sugitani et al., 2002) as markers. Strikingly, the overall number of Cux1-positive cells was diminished by 15% (Fig. 4A-D′,K; *P*<0.05) in Raldh2cKO mutants. In contrast, the overall numbers of Brn2-positive cells showed a 15% significant increase on rostral sections (Fig. 4A,B,E,F,L; *P*<0.05), and a tendency to an increase on more caudal sections (Fig. 4A′,B′,E′,F′,L), in the mutants. The cell density of the layers was evaluated and was unchanged (data not shown). These data further support a role for RA in proper establishment of projection neuron layers in the cortex.

To refine this analysis, we counted the number of Cux1-positive, Brn2-negative cells, which identify layer IV neurons. Strikingly, this number was reduced by 30% (Fig. 4G,H,M; *P*<0.05) anteriorly and 45% caudally (Fig. 4G′,H′,M; *P*<0.05). This observation led us to examine specifically layer IV neurons labelled by RORβ (Jabaudon et al., 2012). A loss by 20% of RORβ-expressing neurons was observed in mutants anteriorly (Fig. 4I,J,N; *P*<0.05) and by 30% posteriorly (Fig. 4I′,J′,N; *P*<0.05). We assessed apoptotic cell death by performing immunolabelling for activated caspase 3 in Raldh2cKO mutants, and compared them with control littermates. No abnormal apoptosis was detected in mutants at E14.5, E16.5, E17.5 and P4 (Fig. S1A-I).

Altogether, these observations suggest that RA is required to establish a proper cortical layering, with the formation of layer IV being most strongly affected in Raldh2cKO mutants.

### Absence of RALDH2 does not affect cell proliferation in the developing cortex

**Fig. 5.**
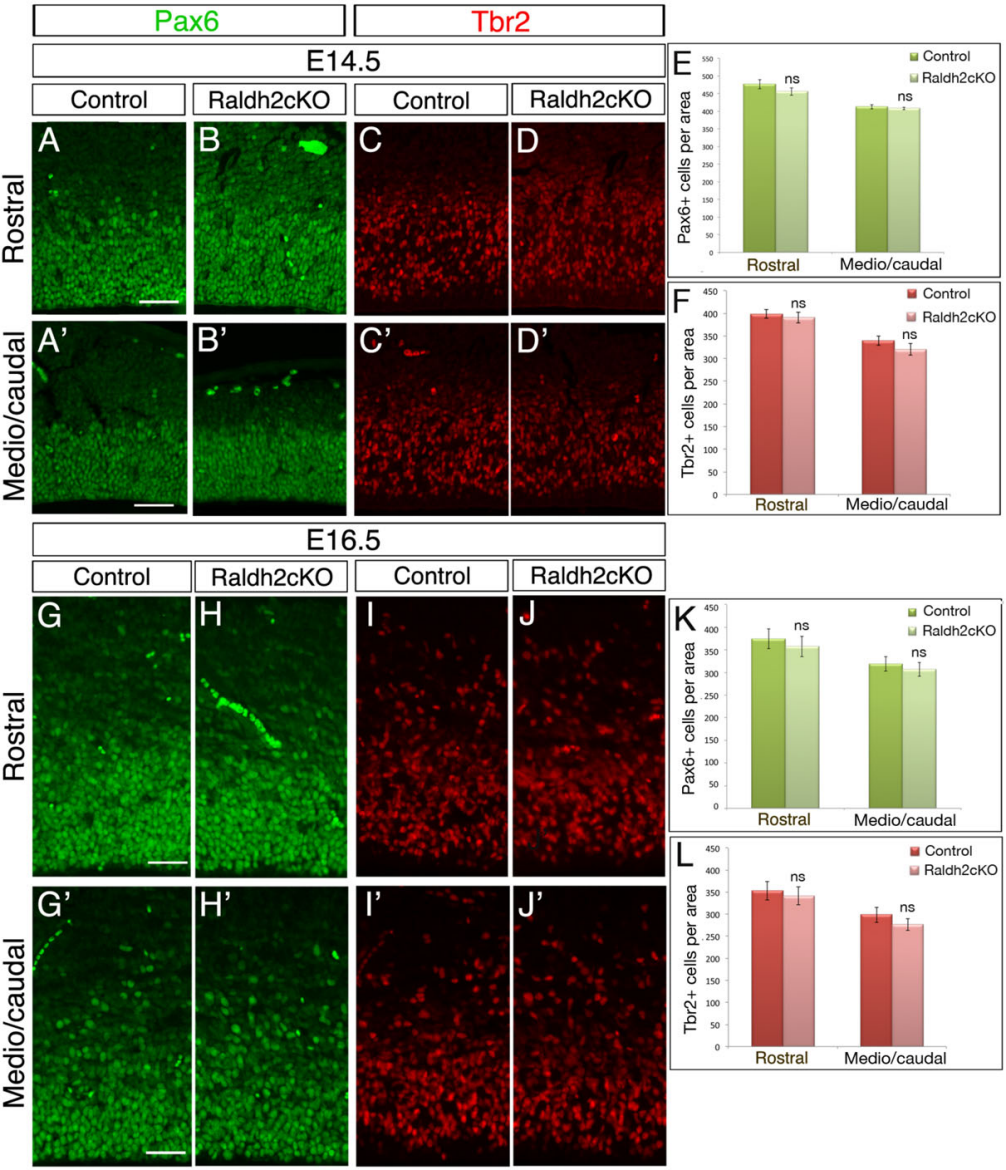
**Raldh2cKO does not affect cortical NPCs.** (A-D′) Immunolabellings on brain sections from E14.5 control and Raldh2cKO animals for Pax6 (A-B′) or Tbr2 (C-D′) at rostral (A-D) and more caudal levels (A′-D′) used for quantification. (G-J′) Immunolabellings on brain sections from E16.5 control and Raldh2cKO animals for Pax6 (G-H′) or Tbr2 (I-J′) at rostral (G-J) and medial levels (G′-J′) used for quantification. (E) Quantification of Pax6-positive cells at E14.5; rostrally: 477.13±13.10 for control and 456±10.48 for Raldh2cKO; caudally: 412.73±6.14 for control and 408.2±3.49 for Raldh2cKO. (F) Quantification of Tbr2-positive cells at E14.5; rostrally: 398.66±9.23 for control and 390.47±11.37 for Raldh2cKO; caudally: 339.47±9.89 for control and 320.13±12.49 for Raldh2cKO. (K) Quantification of Pax6-positive cells at E16.5; rostrally: 374.06±21.71 for control and 357.26±22.71 for Raldh2cKO; caudally: 319±16.42 for control and 306.99±15.28 for Raldh2cKO. (L) Quantification of Tbr2-positive cells at E16.5; rostrally: 353.2±20.93 for control and 341.2±20.23 for Raldh2cKO; caudally: 298.46±16.96 for control and 276.66±13.27 for Raldh2cKO. Data presented as mean±s.e.m.; *n*=5 brains; ns, not significant by two-tailed Student's *t*-test. Scale bars: 50 μm.

In the mouse cerebral cortex, there are two major populations of neural progenitor (NP) cells: radial glial (RG) cells localised in the VZ and expressing Pax6 (Englund et al., 2005), and intermediate neuronal progenitor (INP) cells localised in the SVZ and expressing Tbr2 (Englund et al., 2005; Sessa et al., 2008). Some projection neurons arise from dividing RG cells through a process called direct neurogenesis, but a large number of these neurons arise via an indirect mechanism involving INP cells. To assess whether the imbalanced number of neurons observed in the DL and UL of the cortex in Raldh2cKO mice could be due to an aberrant number of NP cells, we quantified RG cells and INP cells using immunolabelling for Pax6 and Tbr2, respectively, in sections of the cerebral cortex from E14.5 and E16.5 control and Raldh2cKO littermates. The number of Pax6-positive RG progenitors (Fig. 5A-B′,G-H′,E,K) and Tbr2-positive INPs (Fig. 5C-D′,I-J′,F,L) was unchanged at both stages in the Raldh2cKO mutants when compared to controls. These data indicate that absence of RA may not affect the initial number of NP cells, nor the balance between direct and indirect neurogenesis after E14.5.

**Fig. 6.**
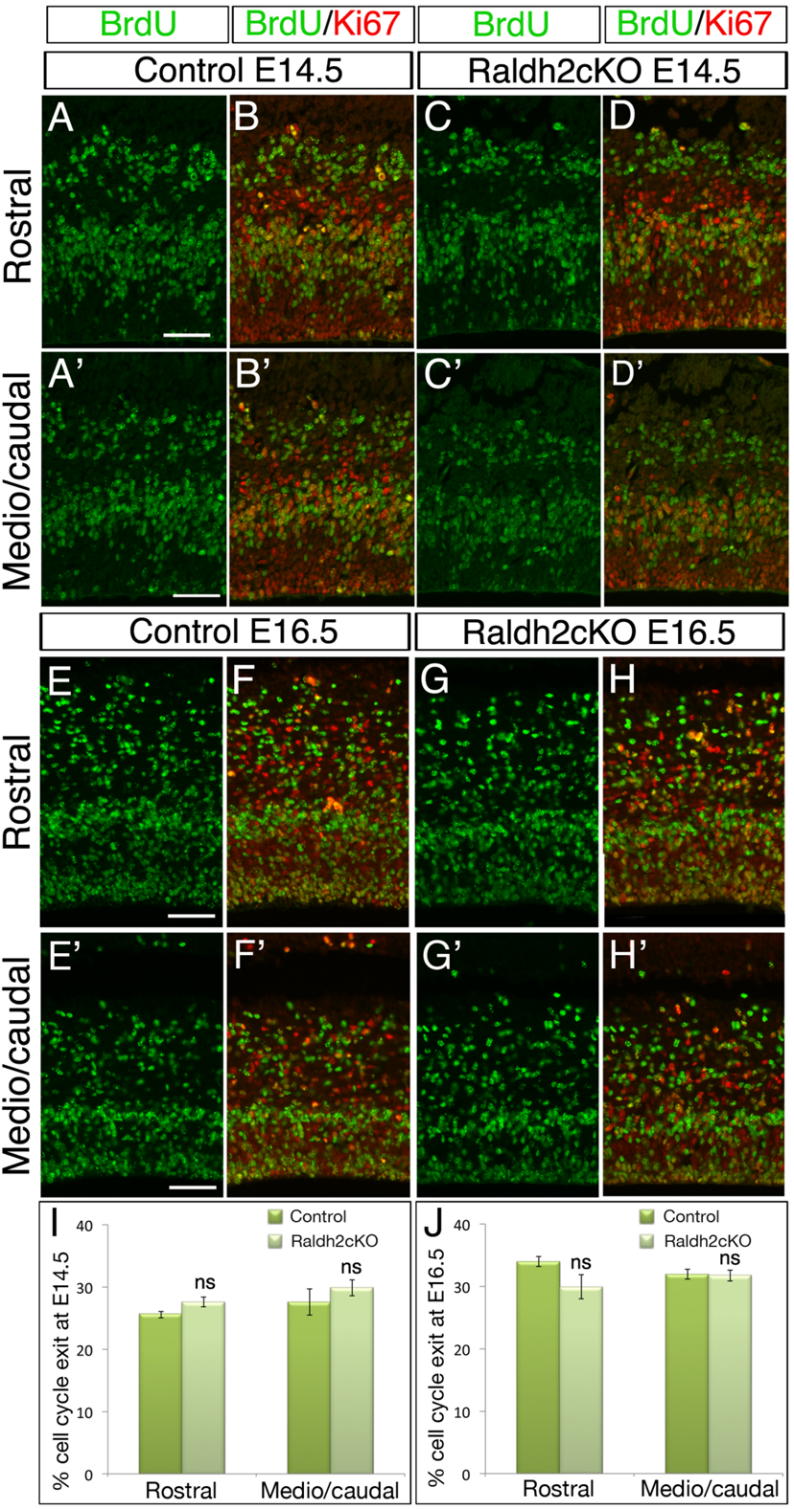
**Raldh2cKO does not affect cell cycle exit of NPCs.** (A-D′) Immunolabellings on brain sections from E14.5 control and Raldh2cKO animals analysed for BrdU (green), which was injected 24 h prior to analysis as a single pulse, and Ki67 (red) at rostral (A-D) and more caudal levels (A′-D′) used for quantification. (E-H′) Immunolabellings on brain sections from E16.5 control and Raldh2cKO animals similarly analysed for BrdU (green) injected 24 h prior to analysis and Ki67 (red). The proportion of cells leaving the cell cycle (BrdU+/Ki67– over BrdU+ cells) is unchanged in Raldh2cKO mutants versus controls. (I) Quantifications at E14.5; rostrally: 25.58±0.54 for control and 27.57±0.75 for Raldh2cKO; caudally: 27.53±0.75 for control and 29.83±1.25 for Raldh2cKO. (J) Quantifications at E16.5; rostrally: 33.92±0.84 for control and 29.92±1.92 for Raldh2cKO; caudally: 31.90±0.74 for control and 31.69±0.92 for Raldh2cKO. Data presented as mean±s.e.m.; *n*=5 brains; ns, not significant by two-tailed Student's *t*-test. Scale bars: 50 μm.

The abnormal distribution of neurons among cortical layers in Raldh2cKO mice could also arise due to an improper fraction of progenitors leaving the cell cycle. Indeed, the observed increase in DL neurons and the decrease in UL neurons could be due to a premature cell cycle exit of NP cells. To evaluate the rate of cells leaving the cycle, we exposed control and Raldh2cKO embryos to a pulse of bromodeoxyuridine (BrdU) 24 h prior to analysis, which was performed at two developmental stages (E14.5 and at E16.5). For this analysis we performed combined BrdU and Ki67 immunolabelling. We quantified the BrdU+/Ki67– cells, corresponding to the cells that were dividing at the time of BrdU injection, but had presumably exited the cell cycle by the time of analysis. These experiments did not reveal any significant difference in the number of counted cells in Raldh2cKO versus control animals, indicating that the progenitors have exited the cell cycle at normal rates to become post-mitotic (Fig. 6), which corroborates the fact that the progenitor pools are not affected (Fig. 5).

Recent studies point to a link between cell cycle length and specification of neocortical NP cells (Arai et al., 2011; Pilaz et al., 2016). We investigated whether changes in the behaviour of cycling progenitors could explain the subsequent defects in neurogenesis observed in Raldh2cKO mice. Immunolabellings performed after a short (1 h) BrdU pulse showed that the numbers of progenitors undergoing S-phase is unchanged in Raldh2cKO mutants (Fig. S2A-F). Furthermore, phospho-histone H3 (PHH3) immunolabellings showed that the M-phase is unaffected (Fig. S2G-J′,K,L).

Taken together, these results suggest that the loss of RA does not affect the number of NP cells nor their proliferating behaviour, i.e. these cells do not appear to differentiate prematurely or in a delayed manner.

### Abnormal specification of newborn neurons to deep layers

**Fig. 7.**
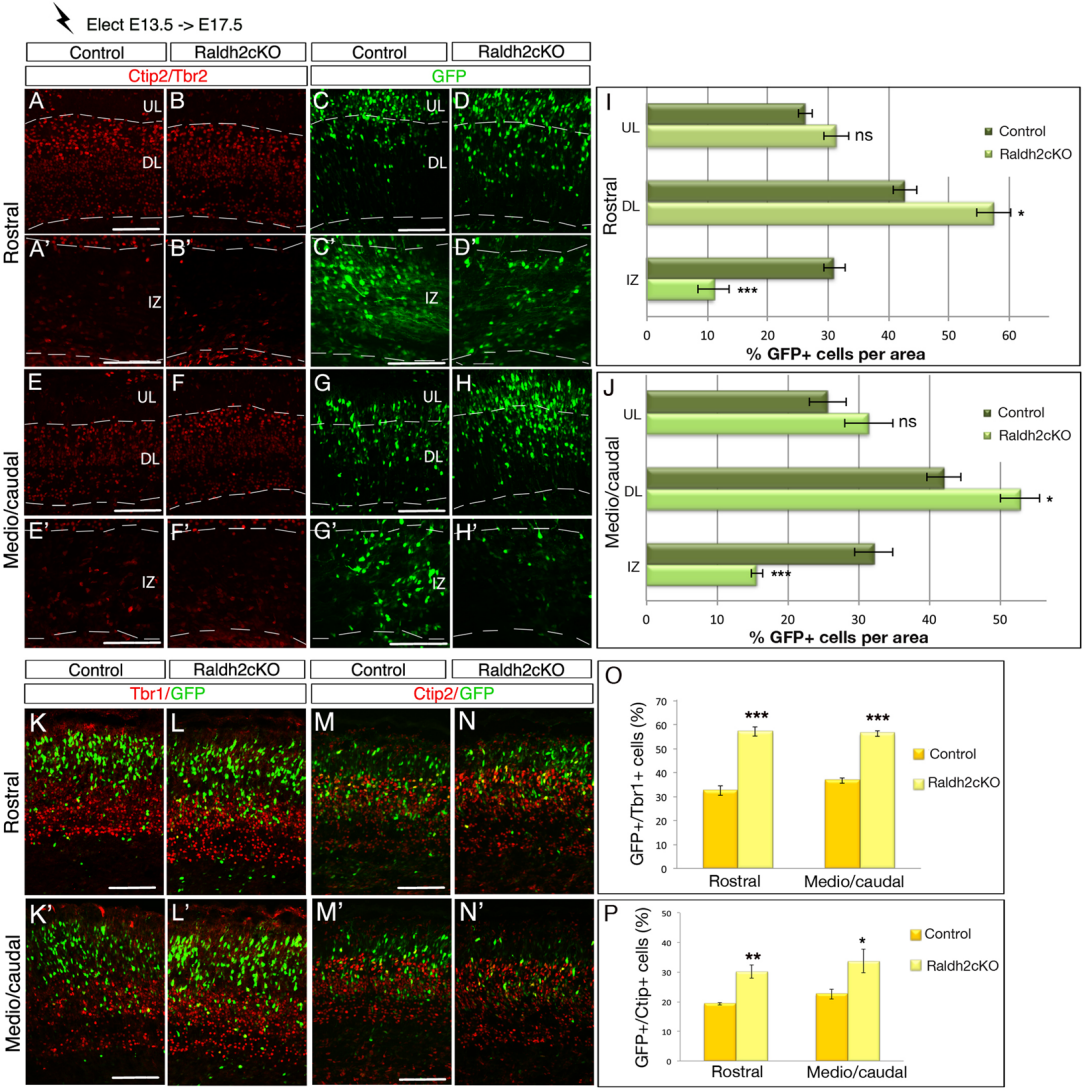
**Lack of RA impairs cell migration of early**-**born cortical neurons.** Brain sections from E17.5 mice electroporated at E13.5 with a GFP reporter construct were analysed for GFP (C-D′,G-H′), and by immunolabelling for Tbr2 and Ctip2 (A-B′,E-F′). Single-color immunolabelling for the two markers help to define the upper layers (UL) and deeper layers (DL) of the cortical plate and the intermediate zone (IZ). (I,J) Histograms depict the percentage of GFP-positive cells per zone (UL, DL and IZ) of the rostral (I) and caudal (J) levels of developing cortex. (K-L′,O) Quantification of GFP-positive cells (green) expressing Tbr1 (red) in the cortical plate of control and Raldh2cKO animals. (M-N′,P) Quantification of GFP-positive cells (green) expressing Ctip2 (red) in the cortical plate of control and Raldh2cKO animals. The histograms (O,P) show the percentage of double-labelled cells over GFP+ cells. Data presented as mean±s.e.m.; *n*=5 brains; *P<0.05, **P<0.01, ***P<0.001 by two-way ANOVA (I,J) and two-tailed Student's *t*-test (O,P). Scale bars: 100 μm.

To further dissect the effects of loss of RA, we assessed the distribution of cortical cells though an approach involving *in utero* electroporation of a green fluorescent protein (GFP)-expressing vector in the VZ of control and Raldh2cKO brains. We performed *in utero* electroporations at E13.5 and E14.5 as these two time points immediately precede the peak of generation of neurons that will form layers V-VI and II-III-IV, respectively (Polleux et al., 1997). We then examined the distribution of GFP-expressing cells at E17.5. Three zones were defined by co-immunolabellings with Tbr2 and Ctip2: the UL (Ctip2–), the DL (Ctip2+), and the intermediate zone (IZ) located between the Ctip2+ and Tbr2+ layers (see Fig. 7A-B′,E-F′).

In brains electroporated at E13.5, we observed at E17.5 a significant increase by 15% in the percentage of GFP-positive cells reaching the DL in Raldh2cKO animals (Fig. 7A-D,E-H,I,J; *P*<0.05). Strikingly, a significant deficit (20%) of GFP-positive cells was observed in the IZ (Fig. 7A′-D′,E′-H′,I,J; *P*<0.001). To strengthen these results, we performed additional birthdating experiments. Pregnant femals were injected with BrdU at E13.5, and the brains were analysed at E17.5. There, BrdU allows detection of cells that underwent the last mitotic division at the time of injection (BrdU being diluted in repeatedly cycling progenitors during the 4-day ‘chase’ period). The analysis of the distribution of BrdU+ cells in the cortical plate corroborates the results obtained with *in utero* electroporation experiments, with an increased percentage of BrdU+ cells in the DL in Raldh2cKO animals (Fig. S3A-D′,I,J).

To assess the effect of RA on differentiation of the DL, we counted GFP+/Ctip2+ cells. Ctip2 is expressed in DL neurons, most of them differentiating before E14.5. Interestingly, in Raldh2cKO brains compared to controls, the percentage of GFP-positive cells expressing Ctip2 was significantly increased from 21% to 32% (Fig. 7M-N′,P; *P*<0.01). Furthermore, we checked if more Tbr1+ neurons are formed at E13.5, i.e. outside of their normal temporal window. For this purpose, we counted GFP+/Tbr1+ cells in brains electroporated at E13.5. Tbr1 is expressed in DL neurons, most of them differentiating at E12.5, but as *Raldh2* is not expressed in dorsal meninges before E13.5 we did not perform electroporation experiments at earlier stages. Our countings showed that the percentage of GFP-positive cells expressing Tbr1 was significantly increased from 35% to 57% in Raldh2cKO mutants compared to controls (Fig. 7K-L′,O; *P*<0.001). This observation corroborates the increase in DL neurons observed at P4 (see Fig. 3C-F′,I,J), and suggests that in absence of RA more neurons differentiate into DL.

In a next series of experiments, we harvested the electroporated embryos two days earlier at E15.5 to investigate if the distribution of GFP-positive cells was already abnormal at an earlier stage of their migration. At this stage, very few newborn neurons have reached the UL (Fig. S4A-D,E-H,I,J). As early as this stage, we observed a significant increase by ∼15% in the percentage of GFP-positive cells that reached the DL at rostral levels (Fig. S4A-D,I) and more caudal levels (Fig. S4E-H,J). A significant deficit of GFP-positive (∼15%) cells was observed in the IZ both rostrally (Fig. S4A′-D′,I) and caudally (Fig. S4E′-H′,J). As our former experiments (Figs 5, 6) indicated that neither the number of progenitors, nor the timing of cell cycle exit was changed in mutants, collectively, the data suggest that in mutants, more neurons born at E13.5 leave the IZ through a facilitated process to reach the DL, thus leading to a reduction of the number of neurons in the IZ in absence of RA and to their misspecification in DL.

### Loss of RA affects upper layer neuron specification by increasing their length of stay in the intermediate zone

**Fig. 8.**
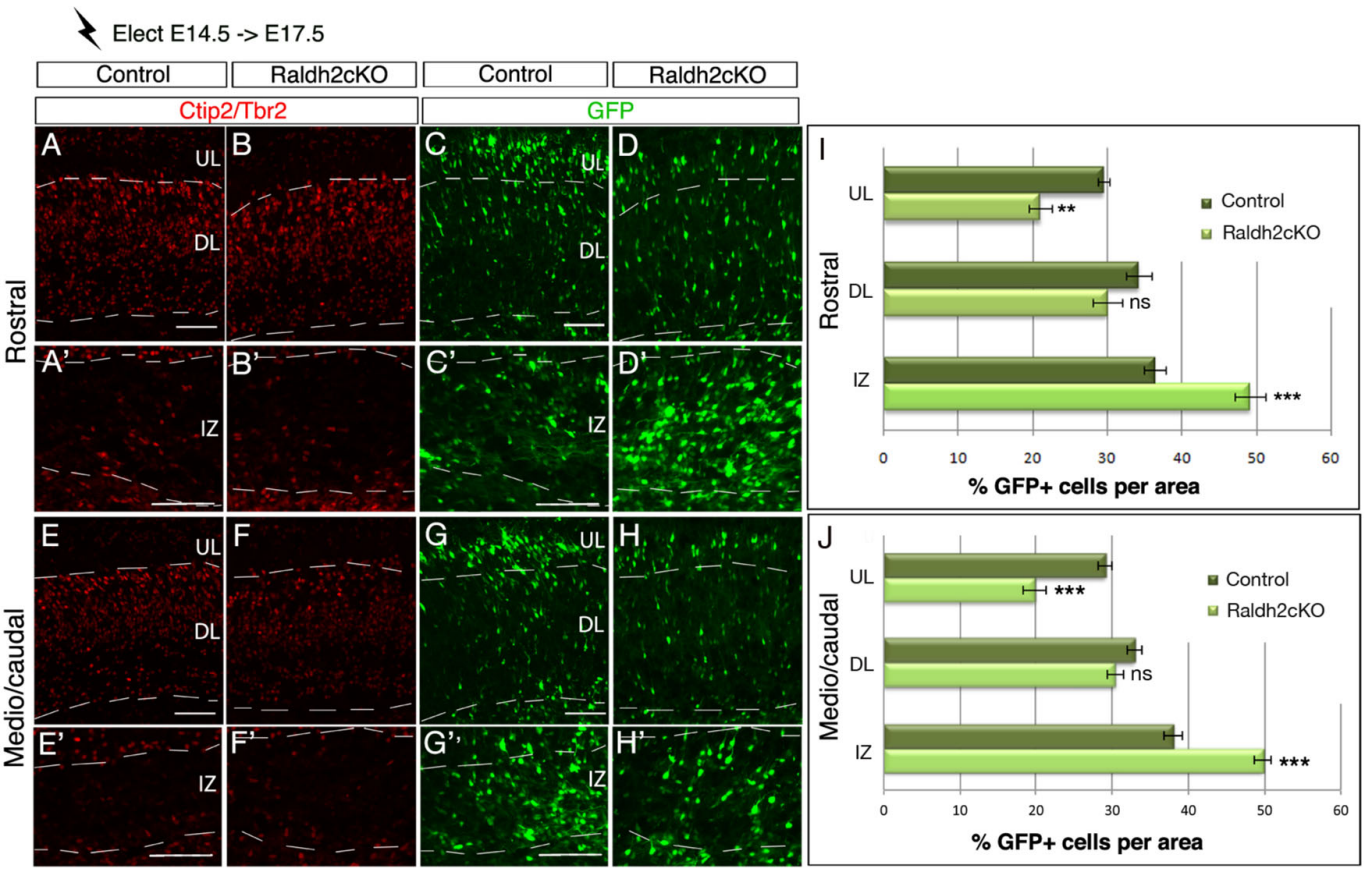
**Lack of RA perturbs cell migration of late**-**born cortical neurons.** Brain sections from E17.5 embryos electroporated at E14.5 with the GFP reporter were analysed by immunolabelling for Tbr2 and Ctip2 (A-B′,E-F′) and for GFP-expressing cells (C-D′,G-H′), at rostral (A-D′) and caudal levels (E-H′) of the cortex. (I,J) Histograms show the percentage of GFP-positive cells in control and Raldh2cKO animals per upper layer (UL), deeper layer (DL) and intermediate zone (IZ) of the developing cortex. Data presented as mean±s.e.m.; *n*=5 brains; **P<0.01, ***P<0.001; ns, not significant by two-way ANOVA. Scale bars: 100 μm.

We next focused on E14.5 electroporations, the time point immediately preceeding the peak of neuronal generation for layers II, III and IV (Polleux et al., 1997). In brains of control animals analysed at E17.5, the majority of GFP-positive cells have reached the UL and DL of the cortical plate (CP), although about a third of the labelled cells were still migrating through the IZ (Fig. 8). Strikingly, in Raldh2cKO mutant brains, the percentage of GFP-positive cells was significantly increased by 12% in the IZ (Fig. 8A′-D′,E′-H′,I,J; *P*<0.001), with a concomitant significant decrease by 9% in the UL (Fig. 8A-D,E-H,I,J; *P*<0.01). The vast majority of GFP-positive cells in the IZ did not express Tbr2, a marker for intermediate progenitor cells, and co-localized with the neuronal marker Tuj1 (Fig. S5), suggesting that those cells are newborn neurons rather than progenitors. In addition, as no ectopic RORβ expression could be detected in the IZ via immunolabelling or *in situ* hybridisation (data not shown), it suggests that those cells have not yet fully progressed through differentiation or are misspecified. Migration of GFP-positive cells to the DL was not significantly affected at this stage in Raldh2cKO mutants (Fig. 8A-D,E-H,I,J). Interestingly, BrdU birthdating experiments revealed abnormal BrdU+ cell distributions in the UL and IZ of Raldh2cKO mutants, consistent with the GFP electroporation data (Fig. S3E-H′,K,L). These observations suggest that disruption of the RA signalling pathway in newborn neurons causes part of them to stall within the IZ and fail to migrate to the CP. Thus, it appears that absence of RA perturbs radial migration of late-born neurons.

To assess whether this accumulation of newborn neurons in the IZ reflects an arrest, rather than just a delay of migration, we repeated the above experiment but instead of collecting the brains at E17.5, these were analysed at E18.5. We observed that the abnormalities in the distribution of newborn neurons that were observed at E17.5 were normalised in the mutant animals at E18.5 (Fig. S6).

Altogether, these observations lead to the conclusion that RA is differentially required for the proper behaviour of early- and late-born neurons. In absence of RA, at E13.5, newborn neurons massively leave the IZ to populate the DL whereas at E14.5, the newborn neurons stall in the IZ and leave it with some delay. These distinct, stage-specific abnormalities could be related to the fact that there are two ways of migration for these neuronal populations: somal translocation is preferentially used by early-born neurons, whereas glial-guided locomotion is used by late-born neurons to migrate longer distances (Nadarajah et al., 2001).

### Newborn neurons destined to upper layers have aberrant morphologies in absence of RA

Electroporation of cortical progenitors at E14.5 in Raldh2cKO mutants leads to an accumulation of post-mitotic (Tbr2-negative and Tuj1-positive) neurons 3 days later in the IZ (Fig. 8; Fig. S5). During normal development, late-born cortical neurons undergo a series of morphological changes to migrate towards the CP. Disruption of these changes will block radial migration. Late-born neurons use glial-guided locomotion to migrate towards UL. First, they acquire in the IZ a transient multipolar morphology by sprouting out mutiple neurites (Noctor et al., 2004). This is followed by the acquisition of a bipolar shape, which will allow their attachment to RG cells and their migration to the CP by glia-guided locomotion (LoTurco and Bai, 2006).

**Fig. 9.**
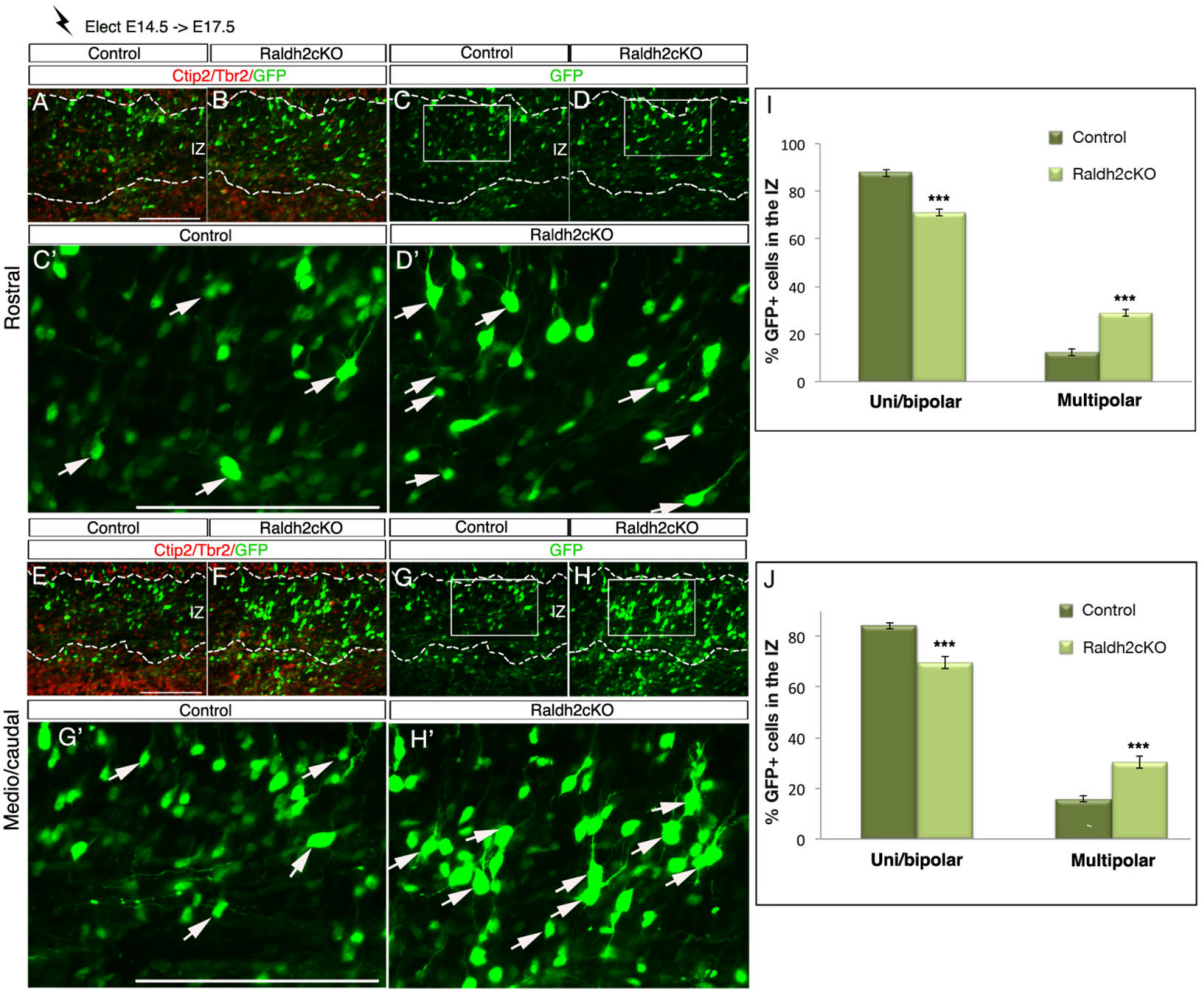
**Aberrant morphology of newborn migrating neurons in Raldh2cKO brains.** *In vivo* electroporation of the GFP reporter was performed on E14.5 cortices, which were harvested at E17.5. (A,B,E,F) Immunolabellings on electroporated control and Raldh2cKO animals for Tbr2 and Ctip2 (red) to define the upper layers (UL), the deeper layers (DL) and the intermediate zone (IZ). (C,D,G,H) GFP signal in electroporated control and Raldh2cKO animals. (C′,D′,G′,H′) Higher magnification views of the areas boxed in C,D,G,H representing the counted areas. More electroporated cells in the IZ exhibit a multipolar morphology in Raldh2cKO mutants (white arrows). (I,J) Histograms showing the distribution of bipolar and multipolar cells in the IZ at rostral (I) and caudal (J) levels. Quantifications. Bipolar cells rostrally: 87.64±1.41% for control and 71.14±1.5% for Raldh2cKO; caudally : 84.17±1.15% for control and 69.70±2.43% for Raldh2cKO. Multipolar cells rostrally: 12.35±1.4% for control and 28.85±1.5% for Raldh2cKO; caudally: 15.82±1.15% for control and 30.29±2.43% for Raldh2cKO. Data presented as mean±s.e.m.; *n*=5 brains; **P<0.01, ***P<0.001; ns, not significant by two-tailed Student's *t*-test. Scale bars: 100 μm.

To check if the glia-guided locomotion could be compromised by abnormal RG processes and/or disruption of the basement membrane, we performed co-immunolabellings with pan-laminin and nestin antibodies at E16.5. No detectable change in morphology of the RG cells and their endfeet were observed in Raldh2cKO mutants, suggesting against the possibility of a disruption of the radial glial scaffolding (Fig. S7). We next performed *in utero* electroporation at E14.5 and examined the morphology of migrating GFP-positive cells in the IZ at E17.5 in control animals and Raldh2cKO mutants. We observed a significant increase in multipolar shaped cells (cells harboring at least three processes) in the IZ of mutants at all brain levels (Fig. 9; *P*<0.001). Accordingly, the proportion of uni/bipolar shaped cells was significantly lower in Raldh2cKO mutants. This suggests that in Raldh2cKO mutants, some of the neurons born at E14.5 and destined to populate the UL retain a multipolar morphology, which will prevent them from correctly migrating to the CP. Our findings strongly suggest a role for RA in promoting multipolar to unipolar/bipolar transition in newborn neurons.

Altogether, our data indicate that in absence of RA, neurons destined to migrate to layer IV (mainly born at E14.5), and some neurons destined for layer II/III, are delayed in their migration towards the UL of the cortical plate. Furthermore, RA may control the transition from a multipolar to a bipolar morphology of late-born neurons, thus influencing their capacity to engage into radial glial-guided migration.

## DISCUSSION

In this study we demonstrate a role for RA during development of the mouse cerebral cortex. We used a conditional gene knockout strategy (Raldh2cKO) ablating the function of a RA-synthesizing enzyme in the developing meninges, and found that this loss-of-function affects cortical layering: the deep layers (V and VI) are increased, whereas layer IV contains fewer RORβ-positive and Cux1-positive neurons. The number of Brn2-positive cells in the upper layers II/III shows a tendency to be slightly increased. These abnormalities are not the consequence of an abnormal establishment or behaviour of the progenitor cell populations. Indeed, we found that loss of meningeal RA does not affect these populations (RG cells and INPs), which are correctly formed and divide to the same extent as in control embryos. Also, the timing of cell cycle exit does not appear to be changed. Interestingly, we showed that the lack of RA affects migration of newborn neurons: in Raldh2cKO mutants, early-born neurons leave massively the IZ to populate layers V and VI, and late-born neurons show a delayed initiation in their radial migration, probably due to an abnormal transition from multipolar to bipolar. One major consequence is on cell specification; in particular, a susbet of RORβ-positive neurons fail to form in the mutant mice.

### RA and cortical neuronal specification

The cortical plate is organised in two populations: the deep layers (DL) V and VI, and the upper layers (UL) II/III and IV. Neurons located in layer II and III connect different cortical areas ipsilaterally or contralaterally. Layer IV is the major input layer as it receives inputs from the thalamus and transmits them to local cortical networks (López-Bendito and Molnár, 2003). Neurons of the DL form connections with subcortical targets including the spinal cord, the pons, the tectum and the thalamus (McConnell, 1995; Molyneaux et al., 2007). During development, the neurons located in a given layer form at the same time, the DL forming first, followed by layers IV, III and II. Progress has been made in identifying genes involved in the specification of progenitors, but little is known on how these various projection neurons are specified. Some studies have shown that neurons are specified at their time of birth (Caviness, 1982; McConnell, 1995), though more recent studies suggest that molecular identities are acquired progressively during postmigratory differentiation (Kwan et al., 2008) even in the first postnatal week (De la Rossa et al., 2013). This progressive refinement implies that some transcription factors are expressed in larger domains encompassing, for example, layer V and VI, are then progressively downregulated via repressive interactions in one of both layers, thus refining the identity of the other layer. This is the case for the DL, where Fezf2 and its downstream effector Ctip2 are expressed in post-mitotic early-born neurons encompassing layer V and VI. Fezf2 and Ctip2 are subsequently downregulated in layer VI by Sox5 and by Tbr1, both factors interacting with Fezf2 (McKenna et al., 2011; Kwan et al., 2008). Thus, Tbr1 is essential to regulate the differentiation of layer VI neurons (Hevner et al., 2001), whereas Fezf2 and Ctip2 are involved in the specification of layer V subcortical projection neurons (Chen et al., 2008; Molyneaux et al., 2005), and fine interactions between those factors progressively define layer V and layer VI (Kwan et al., 2008). In absence of RA, a concomitant increase of layers V and VI is observed. RA could regulate – directly or indirectly – a factor like Fezf2 or Sox5, and the absence of this signal would then perturb the local regulatory loops. Another role of RA could be to regulate an upstream determinant of the DL lineage. This hypothesis is consistent with work showing that the acquisition of a DL fate is actively specified by an environmental signal (Bohner et al., 1997; Desai and McConnell, 2000). Only thereafter, different transcription factors like Tbr1, Fezf2 or Ctip2 interact positively or negatively to define projection neuron subtypes (McKenna et al., 2011). Along these lines, Satb2, which is required to activate a genetic program within early UL neurons and can downregulate DL markers like Ctip2, may be viewed as a ‘master’ determinant of the UL (Britanova et al., 2008; Alcamo et al., 2008), possibly regulated by proper RA levels.

The immature UL are first uniformly labelled by Brn2, whose expression becomes restricted to layers II/III following an upregulation of RORβ in layer IV, via a mutually repressive interaction (Oishi et al., 2016). In absence of meningeal RA, we found that globally all markers are expressed according to their expected position with respect to layering. However, a pronounced effect was observed for RORβ, with a marked decrease of cells expressing this marker in layer IV. Our data suggest that neurons born at E14.5 and contributing to layer IV are delayed in their migration. This delay, by partially preventing RORβ expression, would render Brn2 expression more robust. In other words, RORβ expression might be too weak in some cells to repess Brn2, leading to its ectopic expression. The delay in migration that we observe for neurons born at E14.5 could have consequences on the timing of gene activation due to a longer exposure to signalling factors present in the IZ. For instance, *Satb2* is not activated immediatly after cell cycle exit, but is highly expressed during neuronal migration (Britanova et al., 2008). A delay in migration could diminish the level of Satb2, which in turn would increase locally Ctip2 expression. *Satb2*^−/−^ mutants almost fully lose RORβ expression in layer IV (Alcamo et al., 2008), which is reminiscent of our observation in Raldh2cKO mice, further suggesting that RA may control directly or indirectly the level of *Satb2* expression in UL.

### RA and migration of newborn neurons

During cortical development, early-born neurons use somal translocation to migrate in the DL. After E14, neurons migrate by using another mode of migration called glia-guided migration, which is dependent on the radial glial (RG) fibers. The late-born neurons acquire, in the IZ, a transient multipolar morphology by sprouting out mutiple neurites (Noctor et al., 2004). This is followed by the acquisition of a bipolar shape and attachment to RG fibers, followed by migration in the CP guided by the glia (LoTurco and Bai, 2006). Perturbation of migration is often linked to mutations in genes coding for proteins involved in the function of the cytoskeleton (see Stouffer et al., 2016 for a review). Disruption of the multipolar to bipolar transition will also affect radial migration. Several families of proteins have been shown to be involved in regulating this process (Azzarelli et al., 2014), including members of the Rho GTPase superfamily, like Rnd2, itself controlled by Neurog2 (Heng et al., 2008), or COUP-TFI (Alfano et al., 2011). Also, it has recently been shown that the Wnt/β-catenin signalling pathway needs to be downregulated for multipolar to bipolar transition to occur properly (Boitard et al., 2015). Knockdown of two other genes encoding proteins interacting with microtubules leads to similar phenotypes; LIS1 and DCX RNAi electroporation in rats causes cells with a multipolar shape to accumulate in the IZ (LoTurco and Bai, 2006). Except for Neurog2, which is regulated by RA in the developing spinal cord (Ribes et al., 2008), no link between these genes and the retinoid pathway has been shown. We have observed that in the absence of RA, an abnormal migration due to a delay in the multipolar to bipolar transition of late-born cortical neurons occurs. The molecular events and downstream effectors of RA involved in the control of these processes remain to be identified.

### RA and corticogenesis: how to reconcile with previous studies

It has long been suggested that RA could be a candidate molecule acting as a diffusible signal from the meninges to the developing neuronal layers of the cerebral cortex. This was suggested by the specific expression of *Raldh2*, throughout fetal developmental, in meningeal cells (Smith et al., 2001), while a more recent study has implicated RDH10, the enzyme acting upstream of RALDH2 (Siegenthaler et al., 2009). These authors analysed *Foxc1* mutants, which fail to form forebrain meninges and exhibit a dramatic reduction of neurons and intermediate progenitor cells (with a decreased Tbr2+ population). They observed that an ENU-generated *Rdh10* mutant exhibits a very similiar phenotype to the *Foxc1* mutant, and furthermore, they could rescue the abnormalities of the *Foxc1* mutants by providing maternal vitamin A or RA supplementation (Siegenthaler et al., 2009). To reconcile the differences beween the phenotypes observed in the *Rdh10* mutant (Siegenthaler et al., 2009) and the present Raldh2cKO mice, one should consider the dynamics of expression of the *Raldh* genes in the brain. Indeed, there are two phases through which RA may control dorsal forebrain and cortex development: (1) a phase from E8.5 until E11.5-12.5 corresponding to a local and sequential production of RA first by RALDH2 then RALDH3 in dorsal forebrain neuroepithelium and surface ectoderm, (2) a second phase starting around E13.5 with a new source of RA production corresponding to the meninges. Previous studies have concluded that only very low levels of RA are present in the cortex itself (Chatzi et al., 2011; Luo et al., 2004; Toresson et al., 1999), suggesting that after E13.5 the source of RA is very local and may be diffusing from neighboring tissue, generating a diffusion gradient. The phenotype observed in *Rdh10* mutants (Siegenthaler et al., 2009) may be related to the early phase of RA that would affect the start of neurogenesis and the formation of the pools of progenitors. Indeed, we have observed the same defects by analyzing a *Rdh10^−/−^* mutant generated in the laboratory (Rhinn et al., 2011; C.H., P.D. and M.R., unpublished observations). Thus, we think that the observed defects are not the result of a loss of RA produced by the meninges, but rather by the loss of RA produced earlier by RALDH2 and RALDH3 in the forebrain. On the same line of observation, electroporation of a dominant-negative RA receptor (RAR403) at E14.5, that potently blocks RAR signalling, does not affect progenitor cells, which leave the cell cycle at the same rate as in control animals (Choi et al., 2014). In this case, the effect of RA produced by the meninges and possibly other unknown sources after E14.5 is stopped and the molecular phenotype of the embryos does not ressemble the described phenotype of *Foxc1* or *Rdh10* mutants. In another study, Chatzi and collaborators analysed *Raldh2^−/−^* embryos rescued from lethality by maternal RA supplementation, and did not detect defects in radial expansion or post-mitotic neuron formation (Chatzi et al., 2011). These authors limited their analysis to E14.5, a stage that may be too early to see defects due to a loss of meningeal RA (*Raldh2* being fully expressed in meninges from E14.5). Furthermore, only Ki67 and TuJ1 were used as markers, respectively for proliferating cells and differentiating neurons, which might not have allowed detection of subtle layering or migration defects.

By generating a *Raldh2* genetic ablation from E10.5, we removed the main (if not unique) source of RA in the cortex after E13.5. Our results corroborate those obtained by Choi et al. (2014), who electroporated a dominant-negative RA receptor in cortices, demonstrating that RA is involved in the onset of radial migration by controlling the multipolar to bipolar change in morphology of newborn neurons, and is also involved in controlling neuronal fate, mainly for layer IV neurons. But the phenotype of Raldh2cKO mutants (this study) is not as strong, in that cortices electroporated with the dominant-negative RAR at P4 layers V to III do not maintain their fate but acquire characteristics of layer II (Choi et al., 2014). We may speculate that the weakness of the phenotype of Raldh2cKO is due to the presence of other sources of RA that are blocked by using the dominant-negative approach; there could be RA produced by RALDH1 starting E17.5 in the meninges and/or RA produced by the choroid plexus and present in the circulating cerebrospinal fluid (Alonso et al., 2011). From our study and the study by Choi et al., one can conclude that RA does not affect the progenitors cell population, neither its survival or division rates, but contributes to the regulation of cell migration and cell specification, thus acting as a regulator of cortical layering.

### Conclusion

In conclusion, our data indicate that RA produced by the meninges is required for proper cortical radial cell migration. It is also part of a post-mitotic molecular mechanism contributing to acquisition of proper neuronal identity, and thus to development of a brain with the appropriate connectivities. It is known that inappropriate positioning in the cortical plate leads to neurodevelopmental disorders including lissencephaly, heterotopia and focal cortical dysgenesis (Sarkisian et al., 2008). Also, subtle alterations in neocortical development contribute to alteration in some neural functions, which may lead in human to dyslexia, schizophrenia, epilepsy, and also mental retardation or learning disabilities. Our mutant is a novel, viable animal model showing subte effects that could be causal for one or more of these disorders, and its further behavioural analysis will be of interest in this respect.

## MATERIALS AND METHODS

### Generation of Raldh2cKO, tamoxifen and bromodeoxyuridine (BrdU) treatments

*CMV-βactin-Cre-ERT2* and *Raldh2^flox/flox^* mice were generated and genotyped as previously described (Santagati et al., 2005; Vermot et al., 2006). Tamoxifen (Sigma) was dissolved in pre-warmed corn oil (Sigma) to make a 34 mg/ml solution, which was aliquoted and stored at −20°C. Tamoxifen was administered orally to pregnant females by gavage (10 mg per gavage) at E10.5. Animals were initially difficult to obtain for analyses at postnatal stages, as tamoxifen treatments leads to abortion, female delivery difficulty and absence of feeding (C.H., P.D. and M.R., personal observations; Lizen et al., 2015). To circumvent these effects, we systematically performed caesarian sections at E18, followed by adoption of the delivered pups by foster mothers (BALB/c strain). For BrdU labelling, pregnant females were injected intraperitoneally with a single dose of BrdU (100 mg/kg body weight).

### *In utero* electroporation

Electroporation of the cortex were performed as previously described (Marchetti et al., 2010). Briefly, endofree plasmid DNA solution (*pCAGGS-GFP* plasmid [a gift from Dr J. Godin, IGBMC, Illkirch, France), 5 mg/ml, Qiagen] mixed with 0.05% Fast Green (Sigma) was injected into the lateral ventricles of each embryonic brain at the indicated stages using pulled glass capillaries. The electroporations were performed on whole heads using a Tweezertrode electrode (diameter 7 mm; BTX) connected to a CUY 21 EDIT electroporator (NEPA GENE) with the following parameters: five 45 V pulses, P(on) 50 ms (ms), P(off) 950 ms for E14.5 and five 35 V pulses, P(on) 50 ms, P(off) 950 ms for E13.5. Brains were dissected at the required time point, fixed in 4% paraformaldehyde, cryopreserved in 20% sucrose and sectioned coronally (18 μm thickness, Leica CM3050S cryostat).

### Immunohistochemistry

Brains were sectioned coronally and processed for immunofluorescence or immunoperoxidase. Sections were obtained using a cryostat (14 μm thickness) or a microtome (8 μm, Leica 2035 Biocut), respectively. After antigen unmasking in citrate buffer (0.01 M, pH 6) during 15 min in a microwave oven, slides were blocked with 5% donkey serum, 0.1% Triton X-100 in phosphate-buffered saline (PBS) and incubated overnight with the following primary antibodies: bromodeoxyuridine (BrdU) (1:500, AbD Serotec #OBT0030G), Ki67 (1:300, Novocastra #NCL-KI67P), phospho-histone H3 (1:500, Upstate #05-806); Pax6 (1:300, Covance #PRB-278P); Tbr2 (1:300, eBioscience #14-4875); βIII-tubulin/Tuj1 (1:200, Covance #MMS-435P-100); Tbr1 (1:100, Abcam #ab31940); Ctip2 (1:500, Abcam #ab18465); Cux1 (1:100, Santa Cruz Biotechnologies #sc13024); cleaved caspase-3 (1:200, R&D Systems #NB100-56113); Brn2 (1:1000 Santa Cruz Biotechnologies #sc6029X), RORβ (1:500, Santa Cruz Biotechnologies #sc21354); Calretinin (1:2000, Swant #7699/4); Raldh2 (1:75, Santa Cruz Biotechnologies #sc22591); nestin (1:100; Developmental Studies Hybridoma Bank #rat-401); laminin (1:500, Sigma #L9393). Primary antibodies were visualised by immunofluorescence using secondary antibodies from donkey (1:400, Invitrogen: Alexa Fluor 488 donkey anti-mouse IgG #A-21202, Alexa Fluor 594 donkey anti-rat IgG #A-21209, Alexa Fluor 555 donkey anti-rabbit #A-31572; 1:400, Interchim: Dylight 488 affinipure donkey anti-goat IgG #705-485-147), whereas cell nuclei were identified using DAPI (1:2000). Immunoperoxidase labelling was performed using species-specific biotin-coupled secondary antibodies (1:400, Jackson Laboratories #PK-6101) and detection was performed using a Vectastain Elite ABC Kit (Jackson Laboratories #PK-6101), following the manufacturer's instructions.

### *In situ* hybridisation

*In situ* hybridisation was performed with digoxigenin-labelled probes as previously described (Rhinn et al., 2011). Template DNAs were kindly provided by Dr O. Marin, MRC Centre for Developmental Neurobiology, London, UK (*CxCl12*) and Dr F. Guillemot, the Francis Crick Institute, London, UK (*Reelin*). All expression patterns were documented using a macroscope (Leica M420) or microscope (DM4000B), both connected to a Photometrics camera with the CoolSNAP (v. 1.2) imaging software (Roger Scientific).

### Quantification and statistics

At least four animals of each genotype (control: *Raldh2^flox/flox^; CMV-βactin-Cre-ERT2*^0^ and Raldh2cKO: *Raldh2^flox/flox^; CMV-βactin-Cre-ERT2*^+^) from four different litters were analysed for each experiment. Cell counting was performed on two cortical regions, i.e. at a rostral and more caudal (at the level of the choroid plexus) level. For each region, three adjacent sections were counted. For migration quantification, GFP+ cells were counted in each defined area (UL, DL and IZ) and averaged with the overall number of GFP+ cells through the entire cortical thickness (including UL, DL, IZ, SVZ and VZ). For BrdU birthdating experiments, BrdU+ cells were counted in each defined area (UL, DL and IZ) and averaged with the overall number of BrdU+ cells through the entire cortical thickness. For morphology quantification, multipolar shaped cells (cells harboring at least three processes) and unipolar/bipolar shaped cells were counted in the IZ.

For dual comparison, statistical analysis was performed using two-tailed Student's *t*-test between control and experimental conditions (Microsoft Excel), whereas statistics for multiple comparisons were generated using two-way ANOVA followed by appropriate post-hoc test (GraphPad Prism software version 7). All graphs plot mean±s.e.m. (*P<0.05, **P<0.01, ***P<0.001 for all histograms in figures).

### Animal ethics statement

Animal experimentation protocols were reviewed and approved by the Direction Départementale des Services Vétérinaires (agreement #67-172 to M.R., 67-189 to P.D., and institutional agreement #D67-218-5 for animal housing) and conformed to the NIH and European Union guidelines, provisions of the Guide for the Care and Use of Laboratory Animals, and the Animal Welfare Act.
